# Intrinsically High Thermoelectric Performance in AgInSe_2_ n‐Type Diamond‐Like Compounds

**DOI:** 10.1002/advs.201700727

**Published:** 2017-12-18

**Authors:** Pengfei Qiu, Yuting Qin, Qihao Zhang, Ruoxi Li, Jiong Yang, Qingfeng Song, Yunshan Tang, Shengqiang Bai, Xun Shi, Lidong Chen

**Affiliations:** ^1^ State Key Laboratory of High Performance Ceramics and Superfine Microstructure Shanghai Institute of Ceramics Chinese Academy of Sciences Shanghai 200050 China; ^2^ University of Chinese Academy of Sciences Beijing 100049 China; ^3^ Materials Genome Institute Shanghai University Shanghai 200444 China

**Keywords:** ab initio calculations, diamond‐like compounds, thermoelectric materials, thermoelectric modules

## Abstract

Diamond‐like compounds are a promising class of thermoelectric materials, very suitable for real applications. However, almost all high‐performance diamond‐like thermoelectric materials are p‐type semiconductors. The lack of high‐performance n‐type diamond‐like thermoelectric materials greatly restricts the fabrication of diamond‐like material‐based modules and their real applications. In this work, it is revealed that n‐type AgInSe_2_ diamond‐like compound has intrinsically high thermoelectric performance with a figure of merit (*zT*) of 1.1 at 900 K, comparable to the best p‐type diamond‐like thermoelectric materials reported before. Such high *zT* is mainly due to the ultralow lattice thermal conductivity, which is fundamentally limited by the low‐frequency Ag‐Se “cluster vibrations,” as confirmed by ab initio lattice dynamic calculations. Doping Cd at Ag sites significantly improves the thermoelectric performance in the low and medium temperature ranges. By using such high‐performance n‐type AgInSe_2_‐based compounds, the diamond‐like thermoelectric module has been fabricated for the first time. An output power of 0.06 W under a temperature difference of 520 K between the two ends of the module is obtained. This work opens a new window for the applications using the diamond‐like thermoelectric materials.

## 1 Introduction

Nowadays, advanced technologies based on high‐performance energy materials have triggered a worldwide attention in response to the world's increasing energy crisis and deteriorating environment. One of the promising technologies is the thermoelectric (TE) energy conversion with its ability of directly converting thermal energy into electricity.^1^, ^2^ Efficient TE conversion requires high performing n‐ as well as p‐type elements (legs) that are assembled electrically in series and thermally in parallel in a module. To ensure long‐term service reliability and stability,^3^ the n‐ and p‐type TE legs should have closely matching physical and chemical properties, such as the chemical composition, melting points, and thermal expansion coefficients. The energy conversion efficiency of a TE material is specified by the dimensionless TE figure of merit *zT* = *S* ^2^ *σT*/(κ_L_+κ_e_). The expression can be viewed as consisting of two distinct contributions: the dominator reflecting the electronic efficiency of a material as expressed via the power factor *S* ^2^σ, where *S* is the Seebeck coefficient and σ is the electrical conductivity, and the denominator representing the heat conduction ability and consisting of the lattice thermal conductivity κ_L_ and the electronic thermal conductivity κ_e_. In order to achieve high TE performance, it is essential to enhance the electronic properties (*S* ^2^σ) and minimize the total thermal conductivity (κ = κ_L_ +κ_e_). However, the transport parameters are closely correlated and interdependent. For example, *S* generally decreases with the increasing carrier concentration, that is, with the increasing electrical conductivity. Moreover, since the electronic thermal conductivity is related to the electrical conductivity via the Wiedemann–Franz law, κ_e_ = *LσT*, it increases with the increasing electrical conductivity. Therefore, it is challenging to simultaneously achieve excellent electronic properties and low thermal conductivity in a given material.

**Figure 1:**
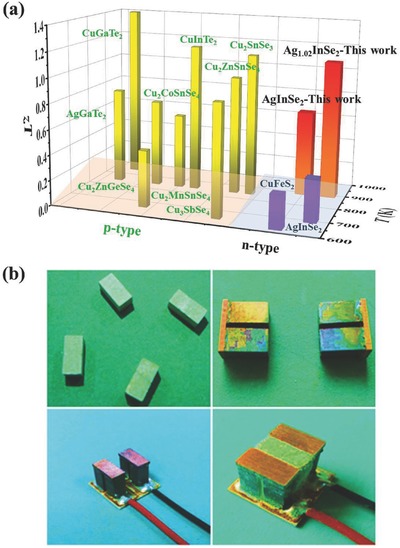
a) Thermoelectric figure of merit *zT* for some typical diamond‐like materials. The yellow columns are for p‐type while the purple and red columns are for n‐type diamond‐like compounds. The data are taken from refs. 9, 10, 11, 12, 13, 14, 15, 16, 17, 18, 19, 20, 21, 22, 23, 24, 25, 26, 27, 28, 29, 30. b) Images of fabricated two‐pair TE module by using high‐performance Ag_0.9_Cd_0.1_InSe_2_ diamond‐like compound as the n‐type leg and Cu_0.99_In_0.6_Ga_0.4_Te_2_ 16 diamond‐like compound as the p‐type leg.

While several families of classical TE materials have been developed and improved, among them SiGe,^4^ Bi_2_Te_3_,^5^ PbTe,^6^ and skutterudites,^7^ they either contain expensive elements or are environmentally harmful. Consequently, there is a worldwide effort to identify and develop new high‐performing TE materials. Recently, many new prospective TE materials have been discovered, and Cu‐based compounds^8^ have generated much interest. Among them, diamond‐like compounds possessing a relatively low thermal conductivity and decent electronic transport properties are especially interesting.^9^, ^10^, ^11^, ^12^, ^13^, ^14^, ^15^, ^16^, ^17^, ^18^, ^19^, ^20^, ^21^, ^22^, ^23^, ^24^, ^25^, ^26^, ^27^, ^28^, ^29^, ^30^ There are about 1000 types of diamond‐like compounds and several of them exhibit very good TE performances. Examples include Cu_2_ZnSn_0.90_In_0.10_Se_4_ with a *zT* of 0.95 at 850 K,^9^ Cu_2_Sn_0.90_In_0.10_Se_3_ with a *zT* of 1.14 at 850 K,^10^ Ag_0.95_GaTe_2_ with a *zT* of 0.77 at 850 K,^11^ Cu_3_Sb_0.97_Ge_0.03_Se_2.8_S_1.2_ with a *zT* of 0.89 at 650 K,^12^ CuGaTe_2_ with a *zT* of 1.4 at 950 K,^13^, ^14^, ^15^, ^16^, ^17^ Cu_2.075_Zn_0.925_GeSe_4_ with a *zT* of 0.45 at 670 K,^18^ CuInTe_2_ with a *zT* of 1.18 at 850 K,^19^, ^20^ Cu_2_TM (TM = Mn, Fe, Co)SnSe_4_ with a *zT* of 0.7 at 850 K,^21^ and many others.^22^, ^23^, ^24^, ^25^ These exciting results make the diamond‐like compounds a new and prospective class of TE materials. However, all the above diamond‐like compounds are p‐type materials. To date, only two examples of n‐type diamond‐like compounds have been reported on and both of them show a rather poor performance: AgInSe_2_ with a *zT* of 0.34 at 724 K^26^, ^27^ and Cu_0.92_Zn_0.08_FeS_2_ with a *zT* of 0.26 at 630 K^28^, ^29^, ^30^ (as shown in **Figure** **1**a). Therefore, the lack of high‐performance n‐type diamond‐like TE materials has, so far, impeded the construction of efficient TE modules based on these compounds.

In this work, we report on n‐type AgInSe_2_ that has an intrinsically low lattice thermal conductivity and its maximal *zT* reaches a value of 1.1 at 900 K. Such high *zT* is mainly due to the ultralow lattice thermal conductivity caused by the Ag‐Se “cluster vibrations” at low phonon frequencies. Furthermore, the average *zT* value of AgInSe_2_ is optimized via substituting Cd at Ag sites to improve the TE performance in the low and medium temperature ranges. In addition, a two‐couple TE module based on these diamond‐like compounds has been fabricated for the first time (as shown in Figure 1b), and has achieved the maximum output power of 0.06 W under a temperature difference of 520 K.

## 2 Results and Discussion

**Figure 2:**
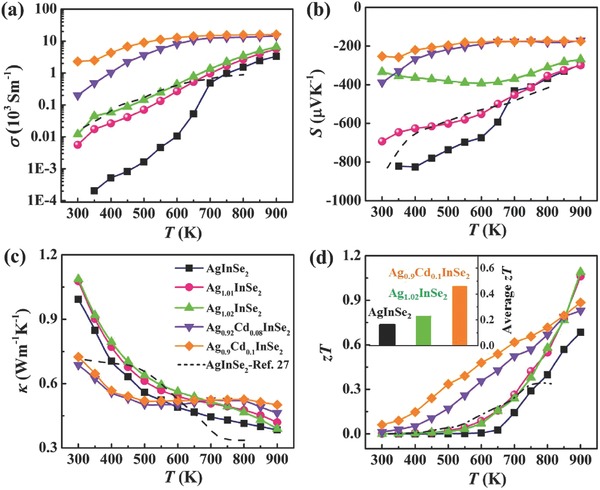
Temperature dependences of a) electrical conductivity σ, b) Seebeck coefficient *S*, c) thermal conductivity κ, and d) *zT* for polycrystalline Ag_1+_ *_x_*InSe_2_ (*x* = 0, 0.01, 0.02) and Ag_1−_ *_x_*Cd*_x_*InSe_2_ (*x* = 0.08, 0.1) compounds. The inset shown in (d) is the average *zTs* for AgInSe_2_, Ag_1.02_InSe_2_, and Ag_0.9_Cd_0.1_InSe_2_ in the temperature range 300–900 K.

AgInSe_2_ is a semiconductor with the band gap of ≈1.19 eV,^31^ which has been extensively investigated for solar energy applications, optoelectronic applications, as well as photoelectrochemical applications.^32^, ^33^ It possesses a typical tetragonal chalcopyrite structure with the space group *I‐42d*, which is derived from the sphalerite structure with Ag/In orderly replacing Zn.^31^ The powder X‐ray diffraction (XRD) patterns of AgInSe_2_ are shown in Figure S1 (Supporting Information). The XRD data match well with the PDF card (#No. 35‐1099) for AgInSe_2_ compounds. The scanning electron microscopy (SEM) results for AgInSe_2_ are shown in Figure S2 (Supporting Information) and demonstrate that a small amount of Ag_2_Se second phase (<3%) exists in the prepared sample. The TE properties of AgInSe_2_ are shown in **Figure** **2**. The electrical conductivity σ of AgInSe_2_ is very low with the values on the order of 10^−1^ S m^−1^ around room temperature and 10^3^ S m^−1^ at 900 K. Correspondingly, the Seebeck coefficient *S* is quite large with a value of −820 µV K^−1^ around room temperature and −295 µV K^−1^ at 900 K. These data indicate that AgInSe_2_ is a typical semiconductor with a very low carrier concentration. This is consistent with our Hall effect measurements, which shows that the carrier concentration of AgInSe_2_ is about 1.3 × 10^13^ cm^−3^ at 300 K. Consequently, the power factor (PF = *S* ^2^σ) of AgInSe_2_ is also very low with a maximum value module 2.92 µW cm^−1^ K^−2^ at 900 K (see Figure S5, Supporting Information). The thermal conductivity for the pristine AgInSe_2_ prepared in our work is as low as 0.99 W m^−1^ K^−1^ at room temperature and decreases to 0.39 W m^−1^ K^−1^ at 900 K, which is close to the theoretical minimum lattice thermal conductivity κ_min_ calculated by the Cahill's model (Equation (S1), Supporting Information).^34^ All these lead to a *zT* of 0.7 at 900 K in AgInSe_2_ although its electrical conductivity is low (as shown in Figure 2d).

The low carrier concentration in AgInSe_2_ suggests that the low *zT* shown above as well as that reported in the literature^26^, ^27^ should have a large room for improvement. In order to change the material's carrier concentration, we thus tried to introduce off‐stoichiometry in the composition. Specifically, we added an excess amount of Ag to AgInSe_2_ to generate interstitial Ag atoms, expecting to increase the density of electrons. The powder XRD patterns for Ag_1+_ *_x_*InSe_2_ (*x* = 0, 0.01, 0.02) are shown in Figure S1 (Supporting Information), and they match well with the PDF card (No. 35‐1099) for the AgInSe_2_ compound. The SEM results for Ag_1.02_InSe_2_ are shown in Figure S3 (Supporting Information) and they too indicate the presence of a small amount of a second phase (Ag_2_Se) in the compound. The TE properties of Ag_1+_ *_x_*InSe_2_ are shown in Figure 2. As we expected, the electrical conductivity σ of Ag_1+_ *_x_*InSe_2_ compounds with excess of Ag has increased to ≈10 S m^−1^ at room temperature, about 1–2 orders of magnitude in comparison to the stoichiometric AgInSe_2_ compound. At 900 K, σ has also significantly increased. The enhancement in σ is mainly derived from the drastically increased carrier concentrations by the excess amount of Ag. The measured carrier concentration at 300 K is 2.5 × 10^15^ cm^−3^ for Ag_1.01_InSe_2_ and 1.6 × 10^16^ cm^−3^ for Ag_1.02_InSe_2_, about 2–3 orders of magnitude enhancement as compared with the stoichiometric AgInSe_2_ compound. The room‐temperature Hall mobility μ_H_ of Ag_1+_ *_x_*InSe_2_ is in the range of 26–68 cm^2^ V^−1^ s^−1^. In contrast, the Seebeck coefficient *S* for the Ag‐excess Ag_1+_ *_x_*InSe_2_ compounds has decreased with the increasing content of Ag throughout the whole temperature range investigated. Around room temperature, the *S* for Ag_1.02_InSe_2_ has greatly decreased to −355 µV K^−1^. In spite of this decrease, as shown in Figure S5 (Supporting Information), the power factor has significantly increased in the entire temperature range. Compared to the stoichiometric AgInSe_2_ around room temperature, the PF for Ag‐excess AgInSe_2_ has increased by about 50 times. Moreover, the maximum PF value of 5 µW cm^−1^ K^−2^ have been reached in Ag_1.02_InSe_2_ at 900 K, an enhancement of about 72% over the value of the stoichiometric AgInSe_2_ compound.

The thermal conductivity κ, on the other hand, is almost unchanged upon introducing an excess amount of Ag in AgInSe_2_, as shown in Figure 2c. The thermal conductivity has a low value of 1.1 W m^−1^ K^−1^ at 300 K and decreases to 0.4 W m^−1^ K^−1^ at 900 K. Combining the significantly improved electronic transport properties with the essentially unchanged and low thermal conductivity, a high *zT* of 1.1 is achieved for Ag_1.02_InSe_2_ at 900 K, an increase of about 62% compared to the stoichiometric AgInSe_2_ compound. The value of *zT* stands as the best n‐type diamond‐like TE material reported so far, and is comparable to the best p‐type diamond‐like TE materials shown in Figure 1a. Having both n‐ and p‐type high‐performance diamond‐like materials available bodes well for fabrication of high‐efficiency TE modules.

**Figure 3:**
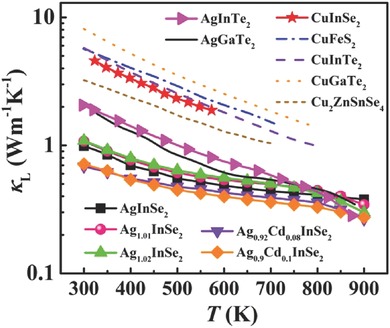
Temperature dependence of the lattice thermal conductivity κ_L_ for AgInSe_2_‐based compounds and other diamond‐like compounds taken from ref. 9, 11, 16, 19, 29, 35, and 36.

Perhaps the most striking feature of AgInSe_2_ is its ultralow lattice thermal conductivity κ_L_. The temperature dependence of κ_L_ for AgInSe_2_ and other typical diamond‐like compounds is shown in **Figure** **3**. The κ_L_ of the stoichiometric AgInSe_2_ compound is about 0.99 W m^−1^ K^−1^ at 300 K, which is much lower than all other Cu‐based diamond‐like compounds, including Cu_2_ZnSnSe_4_ (3.3 W m^−1^ K^−1^ at 300 K),^9^ CuGaTe_2_ (7.4 W m^−1^ K^−1^ at 300 K),^16^ CuInTe_2_ (5.4 W m^−1^ K^−1^ at 300 K),^19^ CuFeS_2_ (5.7 W m^−1^ K^−1^ at 300 K),^29^ and CuInSe_2_ (4.6 W m^−1^ K^−1^ at 300 K).^35^ In particular, as shown in Figure 3, even though the Ag(Ga,In)Te_2_‐based diamond‐like compounds contain a heavy Te element, their lattice thermal conductivity at 300 K is almost twice the value of AgInSe_2_ that has a lighter Se element.^11^, ^36^

**Figure 4:**
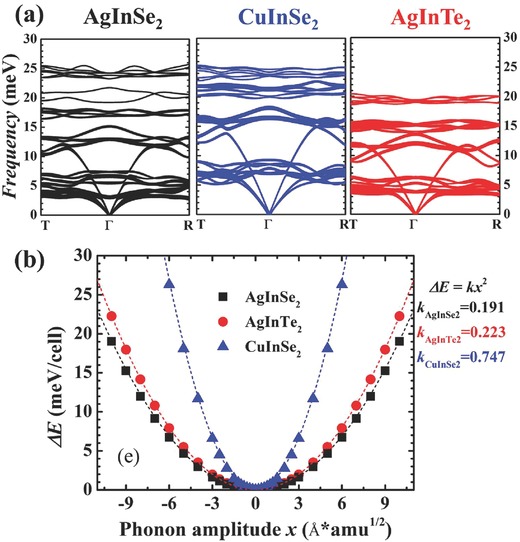
a) Calculated phonon spectra of AgInSe_2_, CuInSe_2_, and AgInTe_2_. The line thickness denotes contributions from Ag or Cu atoms. b) Energy difference Δ*E* as a function of the phonon amplitude for the first optic mode caused by Ag‐Se (or Cu‐Se, or Ag‐Te) clusters. The curves are fitted by a second‐order polynomial Δ*E = kx* 2, and the force constants *k* for these three investigated compounds are labeled.

In order to rationalize this abnormal situation, we performed ab initio lattice dynamics calculations for AgInSe_2_ (see **Figure** **4**a). For comparison, the results for CuInSe_2_ and AgInTe_2_ are also included in Figure 4a. The line thickness in Figure 4a denotes the contributions from Ag or Cu atoms. For crystalline compounds, the low sound velocity, large lattice anharmonicity, and low frequency optic phonons definitely lead to a low lattice thermal conductivity.^37^ Given a phonon spectrum, the sound velocity can be easily obtained by calculating the zone center derivative of acoustic phonon dispersions. The sound velocities for AgInSe_2_, CuInSe_2_, and AgInTe_2_, including longitudinal (*v*_l_), transverse (*v*_t_), and average (*v*_avg._) sound velocities (calculated by Equation (S2), Supporting Information), as well as the Grüneisen parameters (γ) of these three compounds, are listed in **Table** **1**. The experimental sound velocities for AgInSe_2_, CuInSe_2_,^38^ and AgInTe_2_ are also included for comparison. As we expected, CuInSe_2_ possesses the largest sound velocities among the three compounds due to its lightest average atomic mass. The values, however, are lower than those for the CoSb_3_‐based skutterudites (*v*_l_ = 4590 m s^−1^, *v*_t_ = 2643 m s^−1^).^39^ Between the two Ag‐based diamond‐like compounds, our calculation suggests that AgInSe_2_ has a slightly faster *v*_t_ and *v*_l_ than AgInTe_2_, which is confirmed by our experimental data shown in Table 1. However, such a small difference between sound velocities in AgInSe_2_ and AgInTe_2_ obviously cannot account for the much lower κ_L_ in AgInSe_2_ as compared with that in AgInTe_2_. In addition, the Grüneisen parameters obtained either from ab initio lattice dynamics calculations or from measured sound velocities for AgInSe_2_ and AgInTe_2_ are close to each other, and therefore cannot explain the large difference in κ_L_ between the two compounds.

**Table 1:** Sound velocities and Grüneisen parameters (γ) for AgInSe_2_, CuInSe_2_, and AgInTe_2_ diamond‐like compounds obtained from ab initio lattice dynamics calculations and experimental measurements. Equations (S3) and (S4) (Supporting Information) are used to calculate γ from the measured sound velocities

	AgInSe_2_		CuInSe_2_		AgInTe_2_	
	Cal.	Expt.	Cal.	Expt.^38^	Cal.	Expt.
*v*_t_ [m s^−1^]	1867	1515	2131	2100	1726	1516
*v*_l_ [m s^−1^]	3430	3296	4060	3770	3239	3159
*v*_avg._ [m s^−1^]	2082	1707	2383	2338	1928	1704
γ	1.78	2.27	1.83	1.63	1.71	2.13

The most distinct feature of AgInSe_2_ regarding its lattice dynamics is the low‐frequency optic phonons, which cover the frequency range from 3.0 to 7.4 meV (see Figure 4a). These low‐frequency optic phonons can introduce additional phonon scattering channels and scattering rates, or phenomenologically, the resonant scattering that impedes the normal transport of acoustic phonons with similar frequencies, leading to an extremely low heat conduction.^37^ Similar phenomena have also been observed in many other compounds with low lattice thermal conductivity, such as filled skutterudites, Cu_2_Se, and α‐MgAgSb.^7^, ^40^, ^41^ As shown in Figure 4a, the low‐lying optic phonons are also observed in the phonon dispersions of AgInTe_2_. However, the lowest‐lying optic phonons appear already at 3 meV in AgInSe_2_ but at a higher frequency of 4 meV in AgInTe_2_. Furthermore, the range of low‐lying optical phonons in AgInSe_2_ is wider covering 3.0–7.4 meV than that in AgInTe_2_ where it covers the range of 4.0–6.3 meV. These features suggest that the low‐lying optic phonons in AgInSe_2_ not only scatter the acoustic phonons of lower frequencies but also scatter the acoustic phonons over a wider range of frequencies. Therefore, the wider scattering channels with lower frequencies are the main reason for the lower κ_L_ observed in AgInSe_2_ than in AgInTe_2_.

In order to further understand the origin of the low‐frequency optic phonons in AgInSe_2_, the phonon animation analysis was performed (see such animation in the Supporting Information). Interestingly, we observed collaborative motions of Ag‐Se clusters (red and yellow balls), while In atoms (green balls) stand still. This strongly suggests that the interaction between Ag and Se is quite strong, while the interaction between the Ag‐Se cluster and In is quite weak in AgInSe_2_. Generally, the interactions between the neighboring atoms can be semi‐quantitatively evaluated by the force constant (*k*) derived from the calculated potential energy variation (Δ*E*) as a function of the phonon amplitude (*x*).^42^ A smaller *k* usually indicates a weaker interaction and thus a larger space for the motion. Figure 4b shows the calculated potential energy variation (Δ*E*) as a function of the phonon amplitude (*x*) for the first optic mode in AgInSe_2_, which is mainly contributed by the Ag‐Se clusters. The Δ*E* versus *x* curve can be well fitted by the second‐order polynomial with Δ*E* = *kx* 2 and *k* = 0.191. Likewise, Figure 4b also shows the Δ*E* versus *x* curves and the corresponding *k* values for AgInTe_2_ and CuInSe_2_. Clearly, AgInSe_2_ has the lowest *k* value among these three compounds, proving that the Ag‐Se clusters have the weakest interactions with the neighboring In atoms. Such well‐bonded Ag and Se atoms and weakly connected In atoms in AgInSe_2_ lead to special Ag‐Se cluster motions with large mass and thus low‐frequency optic phonons. Similar phenomenon has also been found in S‐ and Se‐filled CoSb_3_ skutterudites, where the strong S(Se)—Sb bonds cause “cluster vibrations” with low frequencies.^43^ Furthermore, since the low‐frequency optic phonons relate to the vibrations of Ag‐Se clusters, it is expected that thus‐caused low κ_L_ can also be found in other diamond‐like compounds containing Ag and Se. For example, the room‐temperature κ_L_ of single‐crystalline AgGaSe_2_ is 1.2 W m^−1^ K^−1^,^44^ much lower than that of AgGaTe_2_ shown in Figure 3a, indicating the generality of the findings in this work.

**Figure 5:**
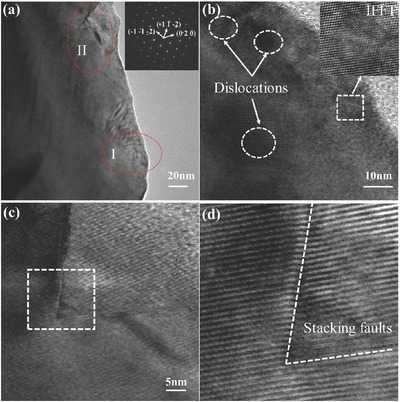
a) Low‐magnification TEM image for stoichiometric AgInSe_2_. The inset is the electron diffraction pattern. b) HRTEM image for area I shown in (a). The inset is the inverse fast Fourier transform (IFFT) image for the square area. c) HRTEM image for area II shown in (a). d) IFFT image for the square area shown in (c).

In addition, low thermal conductivity also relies on strong phonon scattering by grain boundaries, impurities, and various kinds of defects. **Figure** **5**a shows a low‐magnification transmission electron microscopy (TEM) image for stoichiometric AgInSe_2_, in which electron diffraction indicates the tetragonal structure. Figure 5b,c are high‐resolution transmission electron microscopy (HRTEM) images for the red ellipse area marked as I and II in Figure 5a, respectively. Several dislocations or imperfections are notable in area I, which are clearly confirmed through the inversed fast Fourier transform (IFFT) image inserted in Figure 5b. Besides, as shown in Figure 5d, the IFFT image for the square area in Figure 5c depicts obvious stacking faults. All the aforementioned dislocations, imperfections, and stacking faults enhance phonon scattering,^6^ which also contributes to the intrinsically ultralow κ_L_ observed in Figure 5a.

The high TE performance in AgInSe_2_ makes it a good candidate to fabricate n‐type legs of TE devices. However, the electron concentration is still too low for an efficient energy conversion in the low and medium temperature ranges. Since it is difficult to enhance the carrier concentration further by introducing more Ag ions (such extra Ag ions would form impurities with no ability to enhance the electron concentration), we have attempted to enhance the concentration of electrons and increase the electrical conductivity at low and medium temperatures by substituting Cd at the Ag sites.

Powder XRD patterns for the Ag_1−_ *_x_*Cd*_x_*InSe_2_ (*x* = 0.08, 0.1) compounds are shown in Figure S1 (Supporting Information). XRD patterns match well with the PDF card (No. 35‐1099) for the AgInSe_2_ compound. There is also a trace of a secondary Ag_2_Se phase detected in SEM‐EDS of the Ag_0.9_Cd_0.1_InSe_2_ compound (see Figure S4, Supporting Information). The temperature dependence of the electrical conductivity σ, the Seebeck coefficient *S*, the thermal conductivity κ, and the figure of merit *zT* for Ag_1−_ *_x_*Cd*_x_*InSe_2_ (*x* = 0.08, 0.1) compounds is shown in Figure 2. The electrical conductivity σ around 300 K has increased by 3–4 orders of magnitude through doping Cd at the Ag sites. Even at high temperatures, the σ values for Cd‐doped compounds are still larger by an order of magnitude than for the stoichiometric AgInSe_2_ compound. Correspondingly, the Seebeck coefficient *S* at 300 K is decreased to a value of −387 and −253 µV K^−1^ in Ag_0.92_Cd_0.08_InSe_2_ and Ag_0.9_Cd_0.1_InSe_2_, respectively. Finally, the PFs for the Cd‐doped AgInSe_2_ compounds are further increased as compared with the Ag‐excess AgInSe_2_ compounds (see Figure S5, Supporting Information). All these features are clearly a result of the much increased electron concentration. In Ag_0.9_Cd_0.1_InSe_2_, the *n* is increased to 2.2 × 10^18^ cm^−3^ at room temperature, about five orders of magnitude higher than the value of ≈10^13^ cm^−3^ for AgInSe_2_ and two orders of magnitude higher than the value of ≈10^16^ cm^−3^ for Ag‐excess AgInSe_2_. In addition, the thermal conductivity of Cd‐doped AgInSe_2_ is also reduced at low and medium temperatures due to the extra point defect phonon scattering by Cd dopants. Especially, the thermal conductivity for Ag_0.9_Cd_0.1_InSe_2_ has been reduced to 0.69 W m^−1^ K^−1^ at 300 K, a decrease of 46% compared to the stoichiometric AgInSe_2_ compound. As a result, the *zT* is more optimized through the whole temperature range. The average *zT* between 300 and 900 K has increased to 0.46, an ≈188 and 92% enhancement as compared to the stoichiometric AgInSe_2_ compound and the Ag‐excess AgInSe_2_ compound, respectively (see the inset in Figure 2d). Figure S6 (Supporting Information) shows the reproducibility test of the electronic transport properties of the Ag_0.9_Cd_0.1_InSe_2_ sample. The data are almost reproducible during three independent runs. Figure S7 (Supporting Information) shows the stability test for the Ag_0.9_Cd_0.1_InSe_2_ sample under the current density of 12 A cm^−2^, which is usually used for the test of superionic TE materials.^45^, ^46^ The relative resistance (*R*/*R*_0_, where *R*_0_ is the sample's initial resistance) for AgInSe_2_ is almost unchanged after about 50 000 s (14 h) test, proving that the Ag_0.9_Cd_0.1_InSe_2_ sample has a good stability under large current.

**Figure 6:**
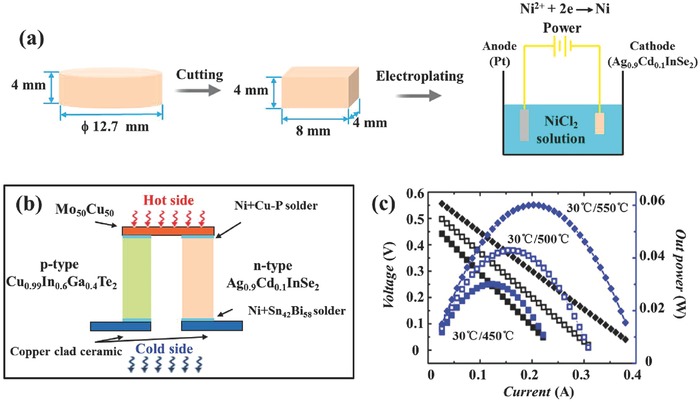
a) Schematic of the fabrication process for diamond‐like modules, including cutting the hot‐pressed cylinder samples into rectangular samples, and the following electroplating process. b) Schematic of the structure of the fabricated diamond‐like module. c) Output voltage (black) and power (blue) as a function of the current for the TE module based on the diamond‐like materials. The temperature difference between the cold side and the hot side of the module is 420, 470, and 520 K, respectively.

The discovered high TE performance in n‐type AgInSe_2_‐based diamond‐like materials makes them suitable, in conjunction with high‐performance p‐type structures, for fabrication of diamond‐like TE modules. Here, we have chosen Ag_0.9_Cd_0.1_InSe_2_ as the n‐type leg and Cu_0.99_In_0.6_Ga_0.4_Te_2_ 16 as the p‐type leg. A two‐couple diamond‐like TE module was successfully fabricated for the first time. As shown in **Figure** **6**a, the cylinder‐shaped samples with a diameter of 12.7 mm and a height of 4 mm were obtained by hot‐pressing (HP) sintering. The ingots were cut into 4 mm × 4 mm × 8 mm rectangles by electrospark wire‐electrode cutting. The two square lateral faces of the samples were electroplated with nickel in a NiCl_2_ solution with an ampere density of 10 mA cm^−2^ for 90 s. Subsequently, the electroplated samples were welded to Mo_50_Cu_50_ alloy blocks with the Cu‐P brazing filler metal (*T*_melt_ ≈ 580 °C) at the hot side and the copper clad ceramic substrates with Sn_42_Bi_58_ (*T*_melt_ ≈ 138 °C) at the cold side. Figure 6b shows schematically the structure of our fabricated diamond‐like TE module. The two π‐pairs were assembled to attain a two‐couple diamond‐like TE module (see Figure 1b). The gap between the two couples was filled with a thermally insulation material to reduce the heat loss. PEM‐2 testing system (ULVAC‐RIKO, Inc.) was used to measure the performance of this module. As shown in Figure 6c, a maximum output power of 0.06 W was obtained with a temperature difference (Δ*T*) of 520 K between the two ends of the TE module. This is the first diamond‐like TE module, clearly showing that it is feasible to fabricate efficient TE devices and perhaps even systems based on the diamond‐like materials. Thus, this work opens the door for applications of diamond‐like TE materials.

## 3 Conclusion

In summary, we have successfully fabricated a series of AgInSe_2_‐based diamond‐like compounds. They are n‐type TE materials. The stoichiometric AgInSe_2_ has very low electron concentration and lattice thermal conductivity as compared with the other diamond‐like materials. Via introducing extra Ag into the material and doping Cd at the Ag sites, the electron concentration has been significantly increased, leading to a much enhanced TE performance with a maximal value of 1.1 at 900 K, comparable to the best p‐type diamond‐like compounds reported before. All the present data strongly suggest that the AgInSe_2_‐based material is currently the best n‐type diamond‐like thermoelectric material. Furthermore, we have succeeded to fabricate a diamond‐like TE module for the first time by using our AgInSe_2_‐based n‐type material. The maximal output power of 0.06 W under a temperature difference Δ*T* of 520 K was obtained, indicating that diamond‐like materials can be a potential candidate for TE applications.

## 4 Experimental Section

Ag_1+_ *_x_*InSe_2_ (*x* = 0, 0.01, 0.02) and Ag_1−_ *_x_*Cd*_x_*InSe_2_ (*x* = 0.08, 0.1) compounds were fabricated by directly reacting Ag (shots, 99.999%, Alfa Aesar), In (shots, 99.999%, Alfa Aesar), Se (shots, 99.999%, Alfa Aesar), and Cd (shots, 99.999%, Alfa Aesar) in sealed silica tubes. The raw materials were weighed out in a stoichiometric ratio and then sealed in silica tubes under vacuum in a glove box. The ingots were obtained by melting the mixture at 1273 K for 12 h, quenched into the icy water and then annealed at 923 K for 5 d. Fine powders were obtained by grinding the ingots in an agate mortar by hand. The powders were then loaded into a graphite die and sintered by hot pressing sintering (MRF Inc., USA) for 30 min at 823 K in vacuum. High‐density pellets (>98% of the theoretical density) were obtained.

The X‐ray diffraction (XRD) analysis (D8 ADVANCE, Bruker Co. Ltd.) was employed to characterize the structure and the purity of the samples. The chemical composition and the microstructure analysis were examined by scanning electron microscopy (SEM, ZEISS Supra 55). The cell parameters were estimated by the Fullprof software (Version February‐2015). The HRTEM images were obtained by JEOL 2100F by using powder samples to investigate the microstructures. The electrical conductivity and the Seebeck coefficient were measured by ZEM‐3 (ULVAC Co. Ltd.) apparatus under helium atmosphere from 300 to 900 K while the thermal diffusivities were measured by the laser flash method (NETZSCH LFA 427) in argon atmosphere. The Neumann–Kopp law was applied to estimate the heat capacity for all samples and the Archimedes method was used to measure the density. The thermal conductivity κ was calculated by κ = *λC*_p_ρ. The experimental sound velocities *v*_t_ and *v*_l_ were obtained by using a ultrasonic measurement system UMS‐100 based on the resonance interference method. The measurements were carried out on the pellet samples with the diameter of 10 mm and the height of 6 mm. The Hall coefficients (*R*_H_) were measured by the Quantum Design Physical properties measurement system (PPMS) from 10 to 300 K with the magnetic field sweeping up to 3 T in both positive and negative directions. The electron concentration (*n*) and the Hall carrier mobility (μ_H_) were calculated by *n* = 1/*R*_H_ *e* and μ_H_ =*R*_H_σ, respectively, where *e* is the elementary charge. The thermal expansion coefficient was measured by Netzsch DIL 402 C.

*Lattice Dynamics Calculations*: First‐principles calculations were performed with Vienna ab initio simulation package (VASP). The generalized gradient approximation (GGA) of the form Perdew–Burke–Ernzerhof and projected augmented wave (PAW) method^47^, ^48^ were adopted. The lattice dynamics calculations for the diamond‐like compounds were carried out by the frozen phonon method, as implemented in the Phonopy package.^49^ 3 × 3 × 3 unit cell (containing a total of 216 atoms in the supercell) was constructed to ensure the convergence of Hellmann–Feynman forces. Accurate convergence criteria, that is, 5 × 10^−5^ eV Å^−1^ for structural relaxation of the unit cell and 10^−7^ eV for static calculation of displaced supercell were used.

*Thermoelectric Modules*: Two n‐p couple module was assembled with n‐type Ag_0.9_Cd_0.1_InSe_2_ and p‐type Cu_0.8_Ag_0.2_In_0.5_Ga_0.5_Te_2_ compounds. Both n‐type and p‐type hot pressing sintered cylinder samples with a size of Φ12.7 mm × 4 mm were processed into 4 mm × 4 mm × 8 mm rectangles with electrospark wire‐electrode cutting. PEM‐2 testing system (ULVAC‐RIKO, Inc.) was employed to evaluate the power output and internal resistance of the module. The electrodes were stable throughout the measured temperature range from 300 to 873 K.

## Conflict of Interest

The authors declare no conflict of interest.

## Supporting information

Supplementary

Supplementary
