# Supplementary material for: Intrinsically High Thermoelectric Performance in AgInSe2 n‐Type Diamond‐Like Compounds
Source: Adv Sci (Weinh). 2017 Dec 18;5(3):1700727. doi: 10.1002/advs.201700727 (PMC5867058; doi:10.1002/advs.201700727)
Supplement: Supplementary file 1 — Supplementary [file ADVS-5-1700727-s002.pdf]

Copyright WILEY-VCH Verlag GmbH & Co. KGaA, 69469 Weinheim, Germany, 2017.

ADVANCED SCIENCE
Open Access

Supporting Information

for *Adv. Sci.,* DOI: 10.1002/advs.201700727

Intrinsically High Thermoelectric Performance in $AgInSe_2$ n-Type Diamond-Like Compounds

Pengfei Qiu, Yuting Qin, Qihao Zhang, Ruoxi Li, Jiong Yang, Qingfeng Song, Yunshan Tang, Shengqiang Bai, Xun Shi,* and Lidong Chen**

Supporting Information

Intrinsically high thermoelectric performance in $AgInSe_2$ n-type diamond-like compounds

Pengfei Qiu, Yuting Qin, Qihao Zhang, Ruoxi Li, Jiong Yang, Qingfeng Song, Yunshan Tang, Shengqiang Bai, Xun Shi*, Lidong Chen**

Physical parameters calculations:

Generally, the minimum thermal conductivity $\kappa_{\min}$ can be obtained according to the Cahill's formula[1]

$$\kappa_{\min} = \frac{1}{2}\left(\frac{\pi}{6}\right)^{\frac{1}{3}} k_B V^{-\frac{2}{3}} (2v_t + v_l), \quad \textbf{(S1)}$$

where V represents the volume per unit atom, k_B is the Boltzmann constant, v_t and v_l are the transverse and longitudinal sound velocities, respectively. The $\kappa_{\min}$ is estimated at 0.39 W m^{-1} K^{-1} for $AgInSe_2$, which is close to the experimental minimum lattice thermal conductivity observed in our work.

Average velocities can be obtained according to Equation[2]

$$v_s = \left(\frac{1}{3} \left[\frac{1}{v_l^3} + \frac{2}{v_t^3} \right] \right)^{-1/3} . \quad \textbf{(S2)}$$

The Poisson ratio (v_p) is calculated by

$$v_p = \frac{1 - 2(v_t / v_l)^2}{2 - 2(v_t / v_l)^2} . \quad \textbf{(S3)}$$

The Gruneisen parameter (γ) is calculated by

$$\gamma = \frac{3}{2}\left(\frac{1 + v_p}{2 - 3v_p}\right) . \quad \textbf{(S4)}$$

Structural characterization

The powder XRD patterns for $Ag_{1+x}InSe_2$ (x=0, 0.01, 0.02) and $Ag_{1-x}Cd_xInSe_2$ (x=0.08, 0.1) compounds are shown in **Fig. S1**. All the patterns indicate strictly the chalcopyrite structure and there are no obvious impurity peaks. As shown in **Table S1**, the lattice parameters and the distortion parameters η (c/2a) for Ag-excess compounds are quite similar to the stoichiometric $AgInSe_2$ compound. After Cd substitution, the lattice parameters are smaller than those for $Ag_{1+x}InSe_2$ (x=0, 0.01, 0.02) compounds and the distortion parameters η (c/2a) are larger than those for $AgInSe_2$.

Figure S1. Powder XRD patterns for (a) $AgInSe_2$, (b) $Ag_{1.01}InSe_2$, (c) $Ag_{1.02}InSe_2$, (d) $Ag_{0.92}Cd_{0.08}InSe_2$, and (e) $Ag_{0.9}Cd_{0.1}InSe_2$. The vertical lines represent the standard PDF card for $AgInSe_2$.

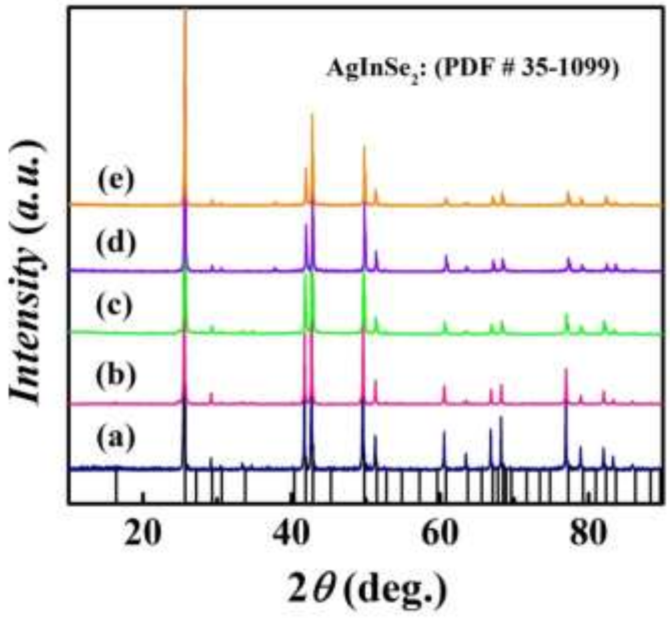

Figure S2 Scanning electron microscopy (SEM) images of the $AgInSe_2$ compound. (a) Backscattered electron imaging map, (b) all elements, (c) Ag, (d) In, and (e) Se mappings.

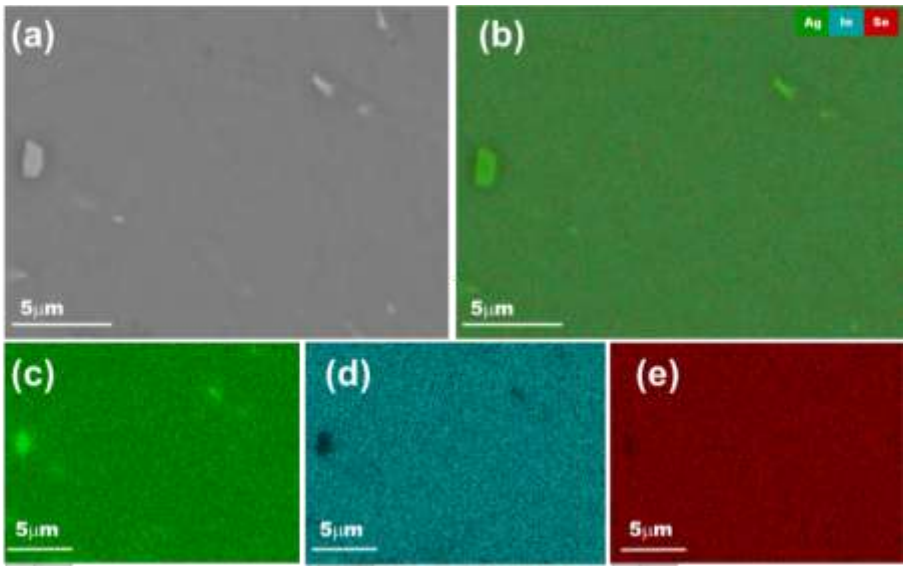

Figure S3 Scanning electron microscopy (SEM) images of the $Ag_{1.02}InSe_2$ compound. (a) Backscattered electron imaging map, (b) all elements, (c) Ag, (d) In, and (e) Se mappings.

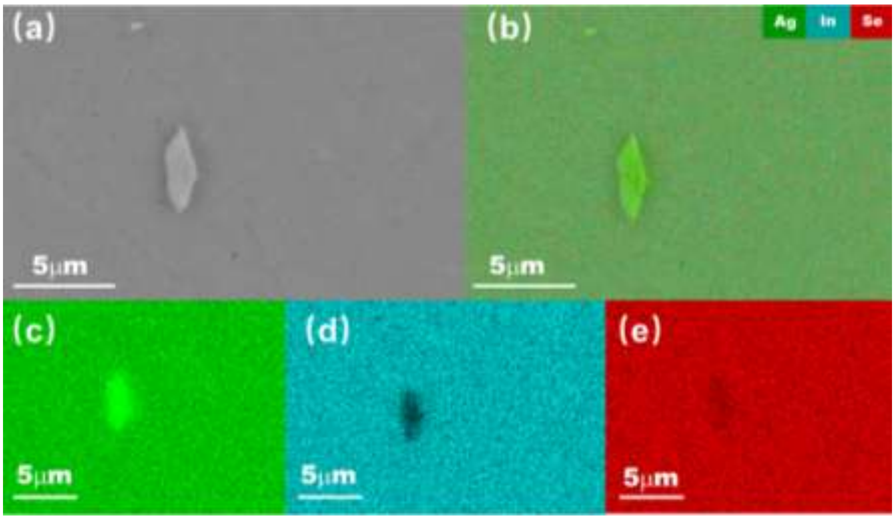

Figure S4 Scanning electron microscopy (SEM) images of the $Ag_{0.9}Cd_{0.1}InSe_2$ compound. (a) Backscattered electron imaging map, (b) all elements, (c) Ag, (d) Cd, (e) In, and (f) Se mappings.

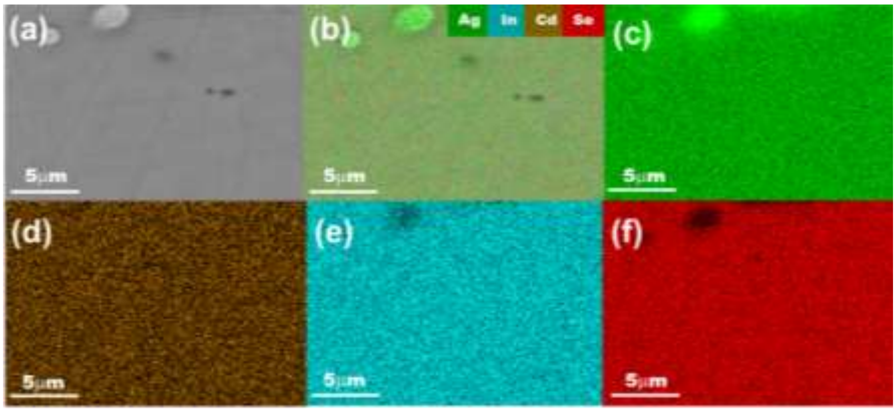

Figure S5. Temperature dependence of the power factor *PF* for polycrystalline $Ag_{1+x}InSe_2$ (x=0, 0.01, 0.02) and $Ag_{1-x}Cd_xInSe_2$ (x=0.08, 0.1) compounds.

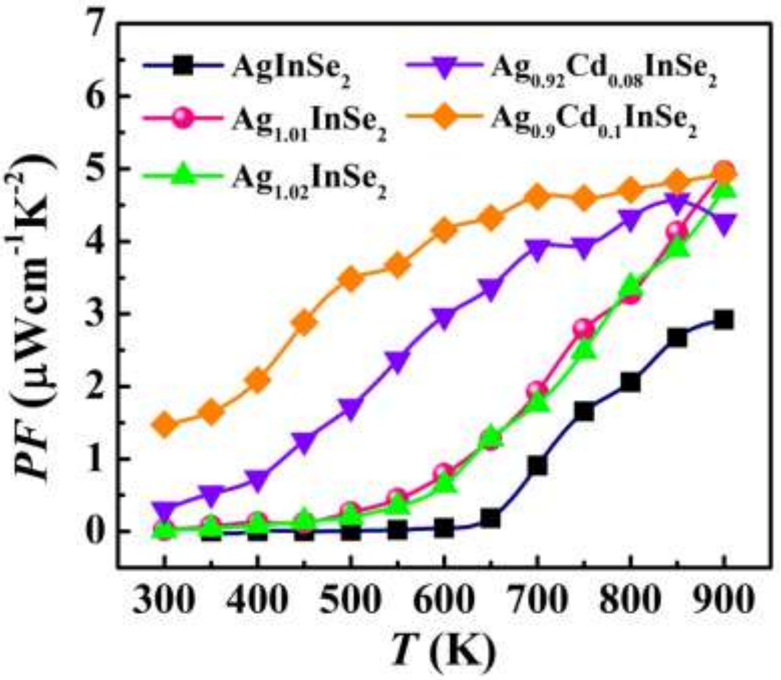

Figure S6. Reproducibility test for the electronic transport properties of the $Ag_{0.9}Cd_{0.1}InSe_2$ sample.

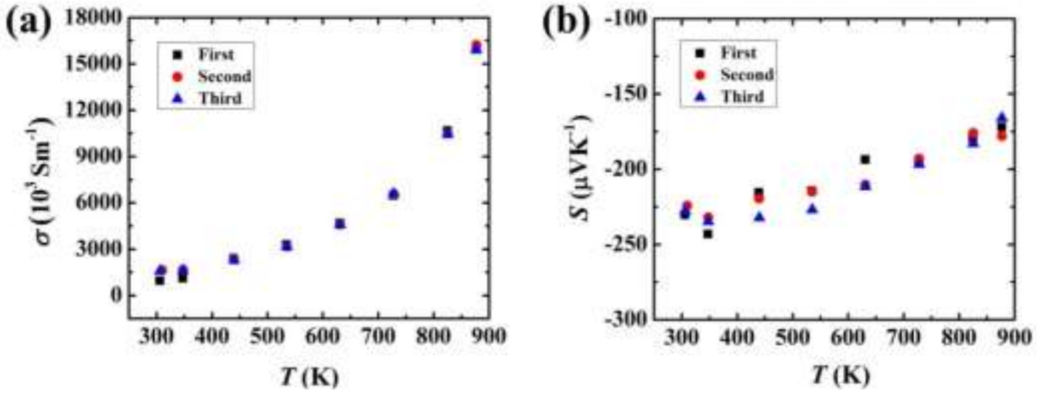

Figure S7. Relative electrical resistance of $AgInSe_2$ as a function of the stress current of the density of 12 Acm^{-2} applied at 650 K for various time durations. The data for Cu_2S at 573 K are taken from Ref. S3 and included for comparson. The measurement details can be also found in Ref. S3.

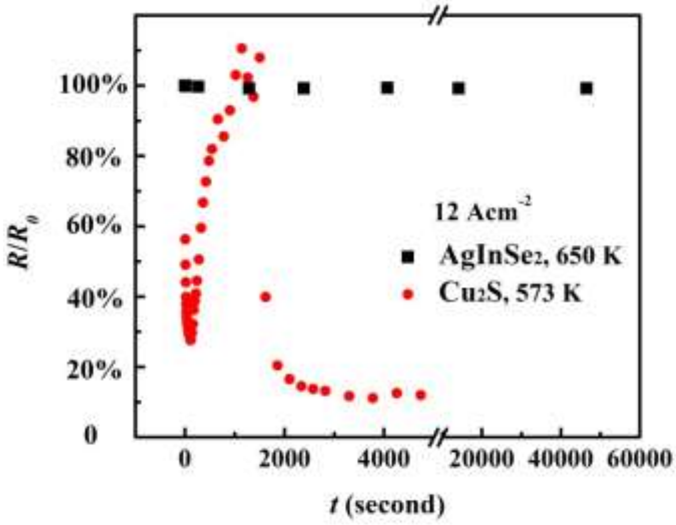

Table S1. Lattice parameters and room temperature TE transport properties for $Ag_{1+x}InSe_2$ (x=0, 0.01, 0.02) and $Ag_{1-x}Cd_xInSe_2$ (x=0.08, 0.1) compounds.

	a (Å)	c (Å)	η ($c/2a$)	n (cm^{-3})	μ ($cm^2\ V^{-1}s^{-1}$)
**$AgInSe_2$**	6.1082(1)	11.7080(2)	0.9584	1.3×10^{15}	25
**$Ag_{1.01}InSe_2$**	6.1067(1)	11.7067(1)	0.9585	2.3×10^{15}	68
**$Ag_{0.92}Cd_{0.08}InSe_2$**	6.1080(1)	11.7090(3)	0.9585	1.6×10^{16}	42
**$Ag_{0.92}Cd_{0.08}InSe_2$**	6.0831(2)	11.7029(4)	0.9619	7.0×10^{17}	27
**$Ag_{0.9}Cd_{0.1}InSe_2$**	6.0866(1)	11.7109(3)	0.9620	2.2×10^{18}	68

Reference

1. D. G. Cahill , S. K. Watson , R. O. Pohl , *Phys. Rev. B* **1992** , *46* , 6131.

2. Y. Xiao, C. Chang, Y. Pei, D. Wu, K. Peng, X. Zhou, S. Gong, J. He, Y. Zhang, Z. Zeng,

L. Zhao, *Phys. Rev. B* **2016**, *94*, 125203.

3. P. F. Qiu, T. S. Zhang, Y. T. Qiu, X. Shi, L. D. Chen, *Energy Environ. Sci*. **2014**, *7*, 4000-4006.
